# Low energy consumption layout of exhibition buildings in Yangtze River Delta region

**DOI:** 10.1038/s41598-024-53439-9

**Published:** 2024-03-11

**Authors:** Wei Zhu, Xinyu Han, Xiaoyu Ying, Yang Tan, Liying Shen, Fanyu Huangfu

**Affiliations:** 1 Hangzhou City University, Hangzhou, China; 2 College of Civil Engineering and Architecture, Zhejiang University, Hangzhou, 310058 China; 3 Zhejiang Engineering Research Center of Building’s Digital Carbon Neutral Technology, Hangzhou, 310015 China

**Keywords:** Energy infrastructure, Civil engineering

## Abstract

The issue of high energy consumption in exhibition buildings has long been a focal point in the field of architectural design. However, current energy consumption assessments for exhibition buildings mainly focus on post-construction evaluations, lacking corresponding guidance during the initial design phase. To address this issue, this study selected 48 well-known exhibition buildings both domestically and internationally as research subjects. Utilizing scatter plot curve fitting, it was discovered that there exists a nonlinear quadratic relationship between the building area of the first floor and the courtyard area. Based on this relationship, four typical layouts were established to match the climatic characteristics of Hangzhou, a representative region in the Yangtze River Delta of China. Taking into account regional architectural features, the study specifically examined the impact of different orientations and window-to-wall ratios on energy consumption levels. The influence of these factors on energy consumption was analyzed using the DesignBuilder software. The results revealed that there exists an optimal window-to-wall ratio for exhibition buildings, with parallel, L-shaped enclosed south-facing courtyards, and U-shaped enclosed east-facing courtyards showing greater energy efficiency. This research provides guidance for designing exhibition buildings that are energy-efficient and foster a harmonious indoor–outdoor relationship.

## Introduction

As urbanization progresses, the size of cities continues to increase. Exhibition architecture, as a type of building with cultural communication purposes, has been increasingly emphasized for its importance, but it has also brought about numerous issues^1^. For example, compared to ordinary buildings, exhibition architecture has higher energy consumption due to the nature of its exhibitions, including long-term mechanical ventilation, the use of heating and cooling systems, continuous use of lighting equipment, and large-sized display screens used for demonstrations. The diversity of interior spaces in exhibition buildings leads to insufficient natural lighting, low utilization of spatial resources, and high heat loss^2^. In comparison to regular office buildings, exhibition architecture presents more complex energy consumption challenges. Firstly, exhibition buildings require large-scale spaces to accommodate a substantial volume of exhibition works. During daily operations, they result in substantial energy consumption. However, because the indoor space of exhibition buildings can exceed 100 square meters with a height of over 15 m, whereas the average human height is about 2 m near the ground, the incongruity between the spatial characteristics of exhibition buildings and human body scale leads to significant energy waste. Secondly, in order to compensate for insufficient natural lighting, exhibition buildings often incorporate courtyard spaces and extensive glass curtain walls to enhance visitors’ leisure experience, which also contributes to heat loss and energy consumption. Therefore, the energy-saving design strategies for exhibition buildings have a certain level of specificity, with the incongruity between spatial characteristics and human body scale, as well as the thermal losses caused by enclosure structures, being both the distinctive features and the challenges of energy-saving design for exhibition buildings. The current situation is due in part not only to the strict physical environmental control requirements for the collections in museum exhibition buildings but also, to a larger extent, to the lack of effective physical environmental control scheme requirements in the initial design phase. This also highlights the importance of overall layout design for energy saving from another perspective. With the formulation of carbon neutral routes, researchers are increasingly focusing on reducing building energy consumption and carbon emissions^3–6^. Studying exhibition architecture as the research object is of significant importance in promoting the realization of carbon neutrality.

To date, many researchers have attempted various approaches to reduce energy consumption in exhibition buildings, yet they still face some challenges. For instance, the Shanghai twenty first century Minsheng Art Museum, despite being a great demonstration of green technologies such as vertical greening, solar photovoltaic power generation, and rooftop gardens after its renovation, encountered issues in subsequent phases, as the high energy consumption and maintenance costs rendered many energy-saving ecological technologies ineffective^7^. However, from a design perspective, it seems that this issue can be addressed. For example, in 2019, Lv et al.^8^ discovered a strong correlation between the energy consumption level, ventilation and lighting efficiency of exhibition halls, and their planar forms, window opening methods, arrangement modes, and orientation when studying exhibition hall forms in severely cold regions. They conducted simulation experiments on case studies such as Jilin Exhibition Center and Liaoning Industrial Exhibition Hall, and proposed optimization strategies that significantly reduced energy waste in exhibition buildings from a design perspective. This viewpoint has garnered support from some researchers as well. Ren et al.^9^ believe that the design stage has a significant impact on building energy efficiency and suggest that nearly 40% of building energy consumption can be addressed during the architectural design phase^10–12^. Therefore, conducting research on exhibition buildings from a design perspective is highly necessary.

Currently, both domestic and international research on exhibition architecture is largely limited to active energy-saving methods such as heating, ventilation, and plumbing^13,14^. In terms of architectural design, China’s current exhibition building design specification JGJ218-2010 is applicable to the design of new, renovated, and expanded exhibition buildings. Architects fully utilize subjective initiative to design an exhibition building within the constraints of this specification, a process that often relies on the subjective experience of the architect. In 2019, Jianguo et al.^15^ used the main pavilion scheme of the Jiangsu International Horticultural Exposition as an example to propose that architects, while respecting the design specifications, should focus on the natural, cultural, and constructive forms to achieve the goal of cultural inheritance and sustainable utilization. Consequently, there is a common phenomenon in architectural design where designers rely on subjective experience for judgment^16,17^, lacking an effective pre-design guidance for low-energy building layout^18^.

The economic and cultural development of the Yangtze River Delta region’s cities is relatively high, with rich cultural lives for residents and significant historical and cultural resources. The rapid development of exhibition architecture in this region, especially in the Jiangsu, Zhejiang, and Shanghai areas, emphasizes outdoor courtyard spaces, which are commonly used for outdoor exhibitions, making spatial comfort of utmost importance. However, the region has a typical hot summer and cold winter climate, with high humidity throughout the year, presenting complex climatic conditions. The summer is sultry, and the winter is damp and cold, creating a pressing need for environmental control^19^. Therefore, there is an urgent need for research on the design of exhibition architecture in the Yangtze River Delta region. Most exhibition buildings in the Yangtze River Delta region incorporate courtyard design^20^. Some researchers have proposed methods to reduce building energy consumption based on courtyard layout. In 2018, He et al.^21^ suggested that courtyards not only improve the microclimate of buildings and reduce indoor energy consumption but also promote interaction and behavioral pattern changes among users. In 2022, Altun et al.^22^ proposed that courtyard orientation and the window-to-wall ratio are important factors influencing energy consumption. Unfortunately, there is still very limited research focused on the layout of exhibition building courtyards and providing design guidance for energy saving.

At the technical level, with the rapid development of computer simulation technology, Witte et al.^23^ have demonstrated the reliability and accuracy of EnergyPlus simulation results. DesignBuilder, incorporating its advantages, addresses the issue of lacking a user interface and has been widely applied in energy consumption simulation research^24^. Based on case studies of parallel buildings, L-shaped buildings, U-shaped buildings, and courtyard buildings, this study combines fitting equations to obtain layout samples. Through DesignBuilder energy consumption simulation and numerical analysis, it provides a reference for designing exhibition buildings with low energy consumption and good indoor-outdoor interaction.

In summary, this study takes Hangzhou, one of the representative cities in the Yangtze River Delta region, as an example to explore the relationship between window-to-wall ratio, orientation, and building energy consumption in different exhibition building layouts. It also aims to address the layout issue at the conceptual design stage. During the early design stage, excellent case studies often provide inspiration and reference for architects. Therefore, from the perspective of architects, this research focuses on 48 well-known exhibition buildings as the study objects. By fitting scatter plots, the relationship between the ground floor area and courtyard area is parameterized to generate typical layout models and explore the influencing factors of energy consumption in exhibition buildings.

This study differs from previous research in three main aspects.

Firstly, this study utilizes regression equations to propose a nonlinear relationship between the ground floor area and courtyard area in exhibition buildings.

Secondly, it comprehensively investigates exhibition building layout forms with lower energy consumption under the summer hot and winter cold climate conditions, representative of the Yangtze River Delta region. This provides guidance for the conceptual design and performance aspects of contemporary fast-paced architectural design.

Thirdly, an energy optimization scheme is conducted using the Liangzhu Culture Museum as a case study, providing empirical validation for this research.

The organization of this paper is as follows: the second section reviews relevant literature and presents the research hypotheses; the third section describes the data collection methods and simulation conditions; the fourth section analyzes the results; the fifth section discusses the findings; and the sixth section presents the conclusion.

## Literature review and research hypothesis

### Literature review

In the field of building energy efficiency, research literature can be broadly categorized into two main types: those focusing on the legal regulations and design standards related to building energy efficiency, and those addressing the theoretical experiments and statistical analyses in the field of building energy efficiency. The following sections will provide a comprehensive review of the research dynamics both domestically and internationally in these two areas:

In terms of legal regulations and design standards for building energy efficiency, the “Law of the People’s Republic of China on the Conservation of Energy” issued in 1997 was the first to incorporate building energy efficiency standards into law. Subsequently, various energy consumption statistical standards for civil buildings were introduced, laying an important foundation for conducting scientific research on building energy efficiency. In theoretical experiments and statistical analyses, research in China started relatively late, with the research content primarily focused on actual measurements. The surveyed building types were mainly office buildings, and there has been limited research on exhibition buildings. The historical development can be summarized as follows: The team led by Tu^25^ conducted the earliest unit area energy consumption surveys and data statistics for government office buildings. In 1990, scholars from Shenyang Jianzhu University conducted field measurements and on-site surveys of energy-related data for buildings in cold regions, laying the foundation for energy consumption research in cold regions. In 2010, Lin et al.^26^ developed a predictive model that can quickly respond to the energy demand of buildings in the design phase, considering the limited known parameters and multiple optimization options in the design stage. By combining Matlab’s genetic algorithm, the optimal solution could be automatically obtained. In 2017, Sun et al.^27^ constructed the GANN-BIM energy-saving design platform by studying the information model of public buildings in severe cold regions. This platform controlled the building form variables to conduct a large number of experiments, thereby obtaining energy-saving design strategies.

In the field of research on low-energy buildings, Western countries, represented by the United States, have an early start. In terms of legal regulations and design standards for building energy efficiency, the Energy Policy Act of 1992, passed by the U.S. Congress, comprehensively addressed energy-related areas, aiming to improve the energy efficiency of various facilities such as civil buildings and electrical equipment. In the realm of statistical analysis, the research organization D&L International Ltd conducted comprehensive statistical analyses of building energy consumption and related data in 1980, publishing the Building Energy Consumption Statistical Yearbook in 2000. From 2006 to 2015, the energy consumption per unit area of public buildings in the United States decreased by 2% annually. In the theoretical research field of energy-efficient design, in 2011, the team led by Austrian architect Ursula Frick first developed the parametric performance design plugin Geco. Suyoto et al.^28^ used the Geco tool to conduct parametric design research based on solar radiation using a public building as an example, proposing a logic for parametric performance design. Subsequently, the development of tools such as Designbuilder and Ladybug + Honeybee^29^ has enabled architects to simulate the performance of parameters such as floor area, orientation, window-to-wall ratio, and envelope structure, leading to widespread application in foreign research on parametric energy-efficient design.

As standards continue to advance, theoretical research on low-energy buildings has seen rapid development. Researchers have placed greater emphasis on passive energy-saving technologies to reduce building energy consumption. Optimizing the window-to-wall ratio is considered an effective and important method^30^. Chi et al.^31^ studied variations in residential orientation and researched the optimal solutions for different orientation angles and window-to-wall ratios (compliant with Chinese building regulations). The results showed that a favorable window-to-wall ratio significantly reduced building energy consumption. Troup et al. suggested that the window-to-wall ratio can help reduce energy consumption in office buildings and demonstrated its potential correlation with building area^32^. Asfour^33^, through studying residential buildings in the Arab region, found a significant correlation between passive energy-saving through window-to-wall ratio control and courtyard layout. Furthermore, the layout of the courtyard and the orientation of the building also exhibited strong correlations with building energy consumption.

In summary, due to differing levels of development among countries, energy conservation goals vary, resulting in significant differences in domestic and international regulations and standards for building energy efficiency. Furthermore, variations in energy consumption calculation tools contribute to differences in energy consumption assessments. OpenStudio is a commonly used energy assessment tool in the United States for energy-efficient design, while in China, self-developed predictive models, EnergyPlus, or other tools based on the core algorithm of EnergyPlus are more prevalent in energy-efficient design. Therefore, it is necessary to discern the research findings of existing parametric energy-efficient designs abroad and establish energy-efficient design strategies applicable to different climatic regions in accordance with China’s specific conditions and energy-saving standards. Regarding variable parameters, the form factor, which describes the building form, is included in China’s energy-saving standards, but its application in building energy-efficient design standards is not yet rigorous enough^34^. Both domestic and international research agree that the building envelope structure affects energy consumption. The window-to-wall ratio, as a crucial indicator for the insulation of the envelope structure, holds significant research value. Additionally, considering the unique nature of courtyard space in exhibition buildings mentioned earlier, orientation affects the layout of courtyards in exhibition buildings, exerting a significant impact on energy consumption. Currently, research on the envelope structure’s window-to-wall ratio and the orientation of courtyards has not yet encompassed exhibition buildings, which has had an adverse impact on reducing energy consumption in this building type.

### Research hypothesis

Based on the findings of previous researchers, we propose the following hypotheses regarding the impact of the window-to-wall ratio on energy consumption, based on four typical layout models in this study: H1: When the window-to-wall ratio of a building is extremely low, a significant amount of energy is required for lighting and heat dissipation. As the window-to-wall ratio gradually increases, the energy required for lighting and heat dissipation decreases. However, when the window-to-wall ratio becomes extremely high, the large area of glass can lead to severe heat loss. Therefore, there may exist an optimal window-to-wall ratio that minimizes energy consumption in building design. H2: During the operational period of a building, the optimal window-to-wall ratio for minimum energy consumption may be related to the building’s floor area. H3: During the operational period of a building, the optimal window-to-wall ratio for minimum energy consumption may be related to the courtyard layout. Based on the optimal window-to-wall ratio for minimum energy consumption, we make hypotheses regarding the influence of orientation indicators on building energy consumption. H4: When the exhibition space avoids excessive daylighting and the courtyard space provides good indoor-outdoor visual interaction, there exists an optimal courtyard orientation layout that minimizes energy consumption. H5: When the layout types of exhibition buildings differ, there are variations in the optimal orientation angles for minimum energy consumption. H6: When the orientation of the courtyard affects building energy consumption, the determining factor is the projected area of windows facing west.

## Research methodology

The energy-saving efforts in many developed countries worldwide started earlier than in China, and their research methods on energy consumption have great reference value for our country. Since the 1960s, developed countries, led by the United States, have developed various energy consumption simulation programs. By the late 1970s, the energy simulation programs BLAST and DOE-2 from the United States, as well as ESP from the United Kingdom, gained global recognition^35^. The fundamental research on building energy consumption mainly focused on basic heat transfer theory and calculating energy loads. BLAST was developed on the basis of Microsoft operating systems and achieved heating energy consumption calculations and tracking of electricity consumption. DOE-2, developed and maintained by LBNL, had fixed format requirements for input files and required a certain level of programming skills in the C language during operation, which limited its widespread adoption among architects. ESP mainly assessed and analyzed factors influencing environmental comfort and building energy consumption. At the same time, Japan also expanded research on building energy consumption to electrical appliances and other power equipment, proposing simulation software for different types of energy conversion systems and developing HASP. It was not until 2001 that multiple scientific organizations in the United States jointly developed EnergyPlus, a robust energy simulation software that replaced BLAST and DOE-2. EnergyPlus combined the heat balance and weighting factor methods and was suitable for the schematic design phase of building projects. It not only helped architects intuitively establish building models and window-to-wall types, but also had simple algorithms that did not require long computation times^36,37^. DesignBuilder, a simulation software based on EnergyPlus, underwent building energy consumption simulation tests by the American Society of Heating, Refrigerating and Air-Conditioning Engineers (ASHRAE). Its annual cumulative heating and cooling load prediction results were compared with predictions from eight other national energy agency-designated energy consumption software, showing good accuracy with a maximum calculation deviation of not exceeding 5.2%. Moreover, DesignBuilder had a powerful built-in database that included most commonly used building materials and their corresponding parameter information^38^. Its strong energy simulation functionality effectively integrated HVAC systems, natural ventilation, building components, and indoor lighting equipment, meeting various computational needs^39^. Therefore, in this study, we simulated energy consumption usingBuilder.

### Research roadmap

**Figure 1:**
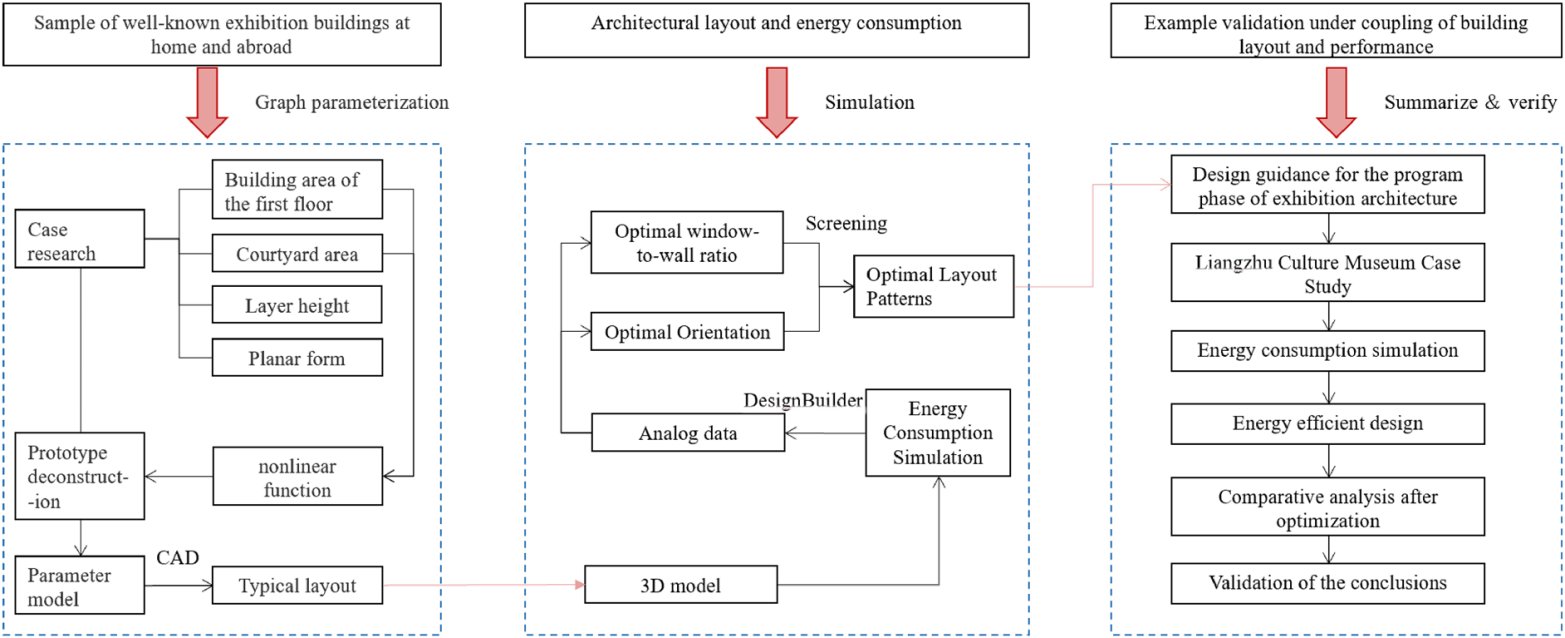
Research roadmap.

The research roadmap of this study is illustrated in Fig. 1. A total of 48 well-known exhibition buildings from both domestic and international sources were selected as the research objects. Data regarding the first-floor building area, courtyard area, floor height, and floor plan were collected and organized. Scatter curve fitting was performed, revealing a non-linear quadratic relationship between the first-floor building area and courtyard area. Based on this functional relationship, four typical layout prototypes were established, and CAD software was used to create drawings. Energy consumption simulations and analyses were conducted using DesignBuilder, focusing on Hangzhou, which represents the typical climate characteristics of the Yangtze River Delta region in China. Taking into account the regional architectural features, particular attention was paid to the impact of different orientations and window-to-wall ratio on energy consumption levels. The optimal layout forms for exhibition buildings were identified, providing guidance for the schematic design phase of exhibition buildings. Lastly, a case study was conducted on the Liangzhu Cultural Museum in Hangzhou to validate the findings by comparing the energy efficiency before and after optimization.

### Collection of research data

To conduct comparative research, this study selected 48 well-known exhibition buildings from both domestic and international sources, including works by Pritzker Prize laureates, architectural websites (such as ArchDaily and gooood), and relevant architectural journals (Table 1). In these buildings, the first-floor area is represented by the gray-white region, while the courtyard area is represented by the yellow region. Due to the complexity of actual layout variations, the layout forms of the buildings can be roughly categorized into five types: parallel, L-shaped enclosure, U-shaped enclosure, square enclosure, and segmented enclosure. The parallel layout mainly exhibits a side-by-side arrangement between the building and courtyard. The L-shaped enclosure layout features a courtyard enclosed on both sides in the form of the letter “L”. The U-shaped enclosure layout showcases a courtyard enclosed on three sides in the shape of the letter “U”. The square enclosure layout presents a courtyard enclosed on all four sides in the shape of the Chinese character “口”. The segmented enclosure layout demonstrates a building with multiple sections enclosing three sides of the building. (The segmented layouts with enclosure on both sides or all four sides have limited examples and are not included in the scope of this study).

**Table 1:** Data of 48 well-known exhibitions building.

Type	Name of the work	First-floor area *X*/m^2^	Courtyard area *Y*/m^2^
Parallel	Ningbo Gang Museum	12,700	9600
(a) Hangzhou History Museum (b) He Art Museum (c) Nezu Museum of Art	Liangzhu Culture Museum	3300	1200
	Hangzhou History Museum	2700	2100
	Scientific Development Outlook Exhibition Center	6770	4800
	Nanjing Museum of Paleontology	4250	1100
	Baohua Institute of Chinese Painting	3700	1100
	Nanjing Holocaust Memorial Hall	14,000	9795
	Beijing Canal Art Museum	1900	600
	He Art Museum	4500	1200
	Guangdong Times Art Museum	2000	1000
	Roca Art Museum, Barcelona	1800	600
	Nezu Art Museum	1500	700
	Conso Modern Art Center	3300	1100
	Long Museum	13,000	5000
	Sol Rock Art Museum	1900	1200
	Yinchuan Art Museum	3890	1400
L-shaped enclosure	Songhu War Memorial Museum	1500	700
(d) Gunma Art Museum	Nanjing Geological Museum	3700	1400
	Gezhong Art Museum	1900	800
	Fort Worth Museum of Modern Art	9240	3140
	Naoshima Museum of Contemporary Art	1200	500
	Gunma Art Museum	2200	1200
U-shaped enclosure	Suzhou Museum	8800	5200
(e) Fei Xiaotong Jiangcun Memorial Hall	New Museum of Nature, Shanghai	11,000	5700
	Suzhou Intangible Cultural Heritage Museum	7600	1500
	Xiahouwen Art Museum	1600	800
	Fei Xiaotong Jiangcun Memorial Hall	1200	1400
	Jinbeier Art Museum	2600	1000
Square enclosures	Zhujiajiao Humanities and Arts Center	900	960
(f) Longyan Cloud Art Museum (g) Han Culture Art Museum	Xuzhou Art Museum	9300	4900
	Huishan Exhibition Center	3200	900
	Han Culture Art Museum	1700	1200
	Haining Museum	2000	600
	Jinyun Museum	2100	600
	Feng Ying-Keng Shi Art Museum	1000	510
	Naruhama-cho Museum of Art	1100	800
	Guggenheim Museum	1600	700
	Kanazawa 21st Century Museum of Art	9515	500
	Longyan Yunduan Art Museum	940	450
	Shanhai Art Museum	1600	700
	Tree Museum of Art	1900	700
	Changjiang Museum of Art	1500	500
Segmented enclosures	Jixi Museum	7000	1500
(h) Muxin Art Museum	Ningbo Museum	5400	1300
	Muxin Art Museum	3060	1200
	Han Pictorial Stone Art Museum	4400	2700
	Hyogo Prefectural Museum of Art	12,807	4200
	Toledo Museum of Art	7000	1000

**Figure 2:**
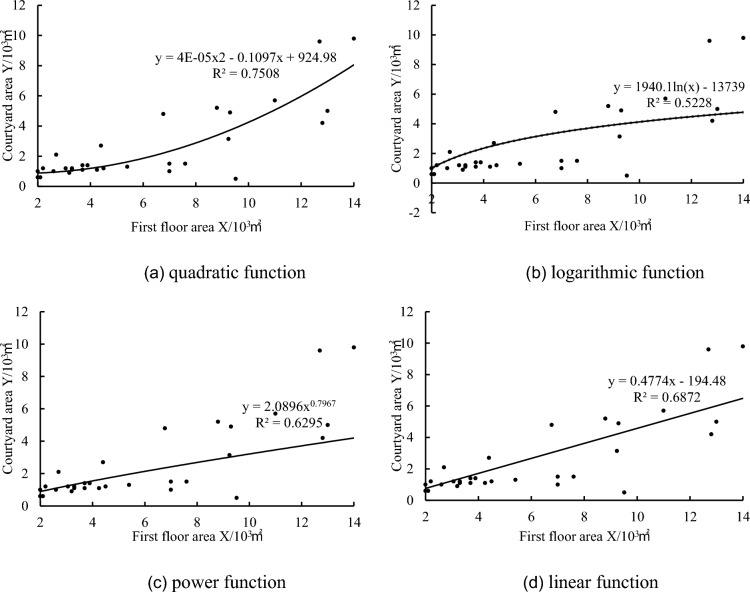
Functional relationship between first floor building area *X* and courtyard area* Y.*

According to Table 1, the first-floor building area X and the courtyard area Y were utilized for regression analysis using Excel software, resulting in four types of functional relationships: quadratic, logarithmic, power, and linear functions (Fig. 2).

According to the “MedCalc Common Statistical Analysis Tutorial”^40^, a coefficient of determination (R^2^) greater than 0.3 is deemed meaningful in the regression equation. Specifically, the R^2^ for the quadratic function is approximately 0.75, for the logarithmic function is approximately 0.52, for the power function is approximately 0.66, and for the linear function is approximately 0.69. This indicates a correlation between the first-floor building area and the courtyard area, with the correlation being most significant in the case of the first-floor building area and courtyard area for the quadratic function, as expressed by the equation:

1 $$Y=4\times {10}^{-5}{X}^{2}-0.1097X+924.98.$$

This non-linear quadratic function is suitable for exhibition buildings with a first-floor building area ranging from 2000 to 14,000 m^2^. Particularly, when the first-floor building area is below 7000 m^2^, the scatter of data points on both sides of the curve is relatively low, indicating that this function accurately represents the relationship between the first-floor building area and courtyard area. Based on this functional relationship, twelve experimental groups were set up for energy consumption simulations to investigate the impact of window-to-wall ratio and orientation on energy consumption in different-sized exhibition buildings. This function has significant implications for the schematic design phase of exhibition buildings.

### Simulation condition settings

The aforementioned architectural cases can be classified into five typical layouts: parallel, L-shaped enclosure, U-shaped enclosure, square enclosure, and segmented enclosure. Since segmented enclosure involves multiple building units and has a relatively low proportion in exhibition building cases, this study focuses on the first four types of single-unit layouts (Table 2). In the diagrams, the white regions represent the buildings, while the grid regions represent the courtyards. According to the “Energy-saving Design Standard for Public Buildings” (GB50189-2015), the window-to-wall ratio for each orientation of a building should not exceed 0.70^41^. Additionally, to meet the requirements of ventilation, heat dissipation, and winter wind protection, public buildings in the Hangzhou area should have a window-to-wall ratio greater than 0.10^16^, with a preference for courtyard layouts facing south, west, and east directions^42^. Moreover, based on the “Daylighting Design Standard for Buildings” (GB/T 50033), to ensure that the exhibition halls have an illuminance standard of 200 lx to 300 lx on the floor and to avoid glare and the adverse effects of direct sunlight on the exhibition experience, the window-to-wall ratio for the west-facing side is uniformly set at 0.10.

**Table 2:** Different layout model settings.

Layout	Model code	Number of layers	Contrast plane	Layout	Model code	Number of layers	Contrast plane
Parallel	A	5F		U-shaped enclosure	C	5F	
L-shaped enclosure	B	5F		Square enclosures	D	5F	

From the above cases, it can be observed that the first-floor area of exhibition buildings is concentrated in the range of 2000 m^2^ to 13,000 m^2^. To further investigate the correlation between the first-floor area and energy consumption, the first-floor area was divided into twelve experimental groups, ranging from 2000 to 13,000 m^2^. Equation (1) was then used to calculate the corresponding courtyard area (Yn) by inputting the first-floor area (Xn), as shown in Table 3.

**Table 3:** Area of 12 experimental groups.

Group	Standard floor area ${X}_{n}$/m^2^	Courtyard area ${Y}_{n}$/m^2^
1	2000	865.6
2	3000	955.9
3	4000	1126.2
4	5000	1376.5
5	6000	1706.8
6	7000	2117.1
7	8000	2607.4
8	9000	3177.7
9	10,000	3828.0
10	11,000	4558.3
11	12,000	5368.6
12	13,000	6258.9

The energy consumption simulation software used in this study was DesignBuilder V6.1. The meteorological parameters of Hangzhou City, a typical city in the Yangtze River Delta region, were selected as the simulation conditions (Tables 4, 5, 6). The activity mode chosen was “Display and Public Areas” under the category “Libraries/Museums/Galleries”. The personnel density, lighting system, ventilation conditions, and indoor temperature all complied with the “Energy-saving Design Standard for Public Buildings” (GB50189-2015) and the “Daylighting Design Standard for Buildings” (GB5003-2013). The air conditioning system employed fan coil units with fresh air systems and air-cooled chillers. The target illuminance for the work surface was set at 200 lx, with a lighting energy consumption of 13 W/m^2^.

**Table 4:** Building model parameters.

Model parameters	
Building type	Exhibition building
Simulated location	Hangzhou City, Zhejiang Province, China
Window to wall ratio	0.1–0.7
Orientation	South, West, East
Number of storeys	5 floors

**Table 5:** Simulation parameters.

Simulation parameters	
Activity template	Display and public areas
Occupancy density (people/m^2^)	0.1497
Heating setpoint temperatures	12 ℃ → 20 ℃
Cooling setpoint temperatures	28 ℃ → 24 ℃
Humidity control	90% → 10%
Power density (W/m^2^)	3.5
Target illuminance (lux)	200

**Table 6:** Exhibition building opening schedule.

Schedule		
WinterDesignDay	Weekdays	9:00 a.m.–12:00 a.m. 1:30 p.m.–5:00 pm.
	Weekends	9:00 a.m.–5:00 p.m.
	Holidays (7 days)	Closed
SummerDesignDay	Weekdays	9:00 a.m.–12:00 a.m. 2:00 p.m.–5:30 p.m.
	Weekends	9:00 a.m.–5:30 p.m.
	Holidays (7 days)	Closed

## Results

### The impact of window-to-wall ratio on energy consumption

**Figure 3:**
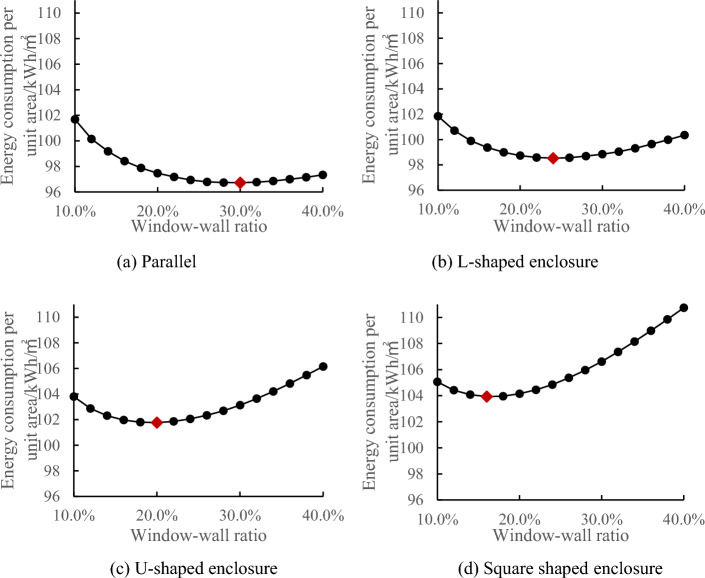
Relationship between window-to-wall ratio and annual energy consumption per unit area for a layout scheme with a standard floor area of 2000 m^**2**^**.**

In order to investigate the variation trend of the minimum energy-efficient window-to-wall ratio among the four types of layouts under different first-floor areas, four layout models were constructed in the DesignBuilder software (Table 7). The first-floor areas ranged from 2000 to 13,000 m^2^, with a total of 12 groups. The simulation calculated the annual energy consumption of the building model when the first-floor area was 2000 m^2^, considering different window-to-wall ratios ranging from 0.1 to 0.7 (with the west-facing window-to-wall ratio fixed at 0.1). The results of the calculation are shown in Fig. 3.

**Table 7:** Model settings in DesignBuilder.

Layout	Model code	Window-to-wall ratio	Model	Layout	Model code	Window-to-wall ratio	Model
Parallel	A	0.1–0.7		L-shaped enclosure	B	0.1–0.7	
U-shaped enclosure	C			Square enclosures	D

From the above figure, it can be observed that when the first-floor area is 2000 m^2^, the annual energy consumption per unit area exhibits a decreasing trend followed by an increasing trend as the window-to-wall ratio increases. Among the four types of layouts, the parallel layout has the highest minimum energy-efficient window-to-wall ratio and the smallest U-shaped layout, with the order of energy consumption being parallel > L-shaped > U-shaped > square. Additionally, from the upward trend of the curve, it can be inferred that the energy consumption does not vary significantly near the minimum energy-efficient window-to-wall ratio, but increases rapidly as the ratio deviates further from the minimum. The square layout shows the largest variation, indicating a significant impact of the window-to-wall ratio on the enclosed structure of the square layout. The simulation results of the 12 experimental groups in the study are consistent with the above conclusions.

**Figure 4:**
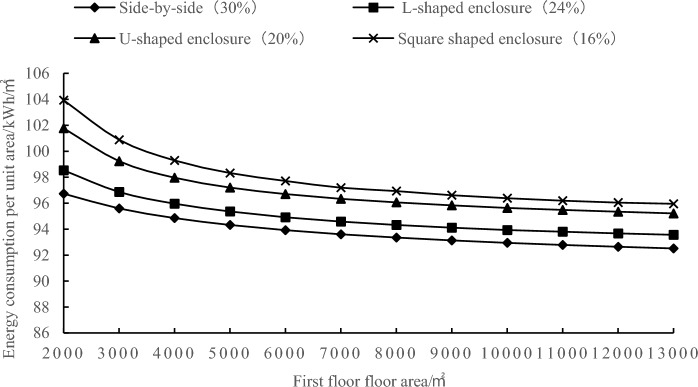
Correlation between standard floor area and energy consumption per unit area.

To further investigate the variation pattern of the minimum energy-efficient window-to-wall ratio when the first-floor area changes, the minimum energy-efficient window-to-wall values mentioned in the above were used for simulation experiments and the simulation results are in Fig. 4. The first-floor area increases the magnitude of the change energy consumption per unit gradually decreases. When the-floor area is ≥ 9000 m^2^, the difference in energy consumption per unit area is below 2 kWh/m^2^, indicating that it has minimal impact on the total annual energy consumption. This suggests that as the first-floor area increases, the change in energy consumption per unit area gradually approaches zero.

**Figure 5:**
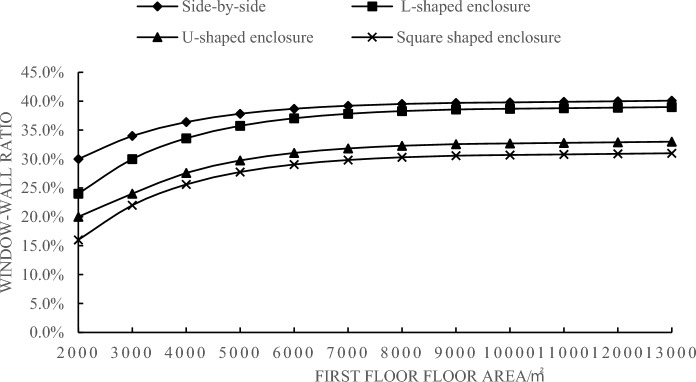
Correlation between first-floor area and minimum energy-efficient window-to-wall ratio.

**Figure 6:**
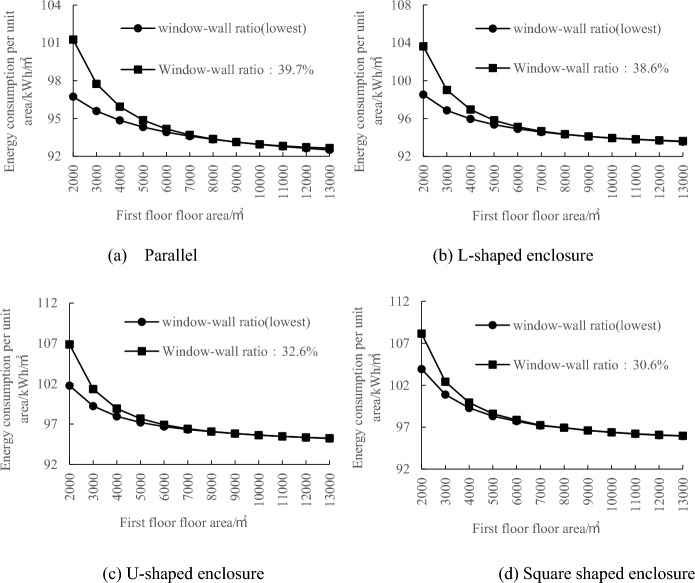
Energy consumption comparison curve between the minimum energy-efficient window-to-wall ratio and the 9000 m^2^ window-to-wall ratio.

As shown in Fig. 5, the minimum energy-efficient window-to-wall ratio increases with the increase in the first-floor area. When the first-floor area is less than 9000 m^2^, the minimum energy-efficient window-to-wall ratio increases rapidly. However, when the first-floor area is greater than or equal to 9000 m^2^, the change in the minimum energy-efficient window-to-wall ratio stabilizes. The difference in annual energy consumption per unit area is below 2 kWh/m^2^, indicating minimal impact on the total annual energy consumption. When the first-floor area is 9000 m^2^, the minimum energy-efficient window-to-wall ratios for the parallel, L-shaped, U-shaped, and square layouts are 39.7%, 38.6%, 32.6%, and 30.6%, respectively. In Fig. 6, the comparison between the curves of these layouts at window-to-wall ratios of 39.7%, 38.6%, 32.6%, and 30.6% with the original minimum energy-efficient window-to-wall ratio is presented. When the first-floor area is greater than or equal to 9000 m^2^, the two curves almost overlap, indicating the existence of a critical value for the minimum energy-efficient window-to-wall ratio in exhibition building design. This value provides crucial references for low-energy emissions reduction during the design phase of exhibition buildings.

In conclusion, after comparing the simulation results of the 12 model groups, it is found that the annual energy consumption per unit area of exhibition buildings exhibits a decreasing-then-increasing trend with an increase in the window-to-wall ratio. This indicates the existence of a minimum energy-efficient window-to-wall ratio, with the parallel layout having the highest ratio and the square layout having the lowest ratio, in the order of parallel > L-shaped > U-shaped > square. The variation in the window-to-wall ratio has a significant impact on the square layout. When the first-floor area is less than 9000 m^2^, the minimum energy-efficient window-to-wall ratio for the four layout types increases as shown in the graph. When the first-floor area is greater than or equal to 9000 m^2^, the minimum energy-efficient window-to-wall ratios for the parallel, L-shaped, U-shaped, and square layouts are 39.7%, 38.6%, 32.6%, and 30.6% respectively. Furthermore, under this window-to-wall ratio condition, the annual energy consumption per unit area remains relatively constant as the first-floor area increases. This window-to-wall ratio is applicable to the walls with different orientations in exhibition buildings, considering energy-saving design specifications and avoiding excessive solar heat gain and glare.

### The impact of building orientation on energy consumption

In order to investigate the energy consumption trends of the four layout types under different orientations, models were built in DesignBuilder software with the following layout settings as shown in Table 8. Since the courtyard side requires a maximum amount of view windows, the window-to-wall ratio for the courtyard-facing walls is set to 0.6, while the window-to-wall ratio for the remaining walls is selected as the optimal value for each layout type mentioned above. Energy consumption simulations were conducted for the 12 experimental groups, each consisting of four layout types. Based on different orientations of the courtyard, a total of 20 plan forms were considered. The simulation results for a first-floor area of 2000 m^2^ are shown in Table 8. From the data presented in the charts and tables, it can be observed that the west-facing layouts of parallel, L-shaped, and U-shaped enclosures exhibit the highest annual energy consumption. Excessive window-to-wall ratios on the west side can significantly contribute to heat loss^43^, thus making it unsuitable to place the courtyard on the west side. The higher energy consumption of the south-tilted west and south-tilted east layouts in the square enclosure is due to the larger window projection area towards the west. Further analysis is required in practical engineering considering massing and breakage design.

**Table 8:** Simulation results of layout with first floor area of 2000 m^2^ under different orientations.

No.	Layout type	Code	Building orientation	Plan form	Window-wall ratio	Total annual energy consumption (kWh)	Energy consumption per unit area (kWh/m^2^)
1	Parallel	Aw	0° due west		0.30	960,193.77	98.63
2		Asw	45° south west		0.30	947,051.16	97.28
3		As	0° due south		0.30	930,263.69	95.56
4		Ase	45° east of south		0.30	945,482.49	97.12
5		Ae	0° due east		0.30	959,247.57	98.53
6	L-shaped enclosure	Bw	0° due west		0.24	968,273.40	99.94
7		Bsw	45° south west		0.24	956,304.59	98.71
8		Bs	0° due south		0.24	949,839.99	98.04
9		Bse	45° east of south		0.24	954,971.98	98.57
10		Be	0° due east		0.24	967,761.72	99.89
11	U-shaped enclosure	Cw	0° due west		0.20	990,846.31	103.19
12		Csw	45° south west		0.20	993,822.34	103.50
13		Cs	0° due south		0.20	995,889.71	103.72
14		Cse	45° east of south		0.20	993,824.26	103.50
15		Ce	0° due east		0.20	990,025.61	103.10
16	Square shaped enclosure	Dsw/Dse	45° S.W./45° S.E.		0.16	1,033,603.38	108.64
17		Dw/Ds/De	0° due west/0° due south/0° due east		0.16	1,032,797.40	108.55

**Figure 7:**
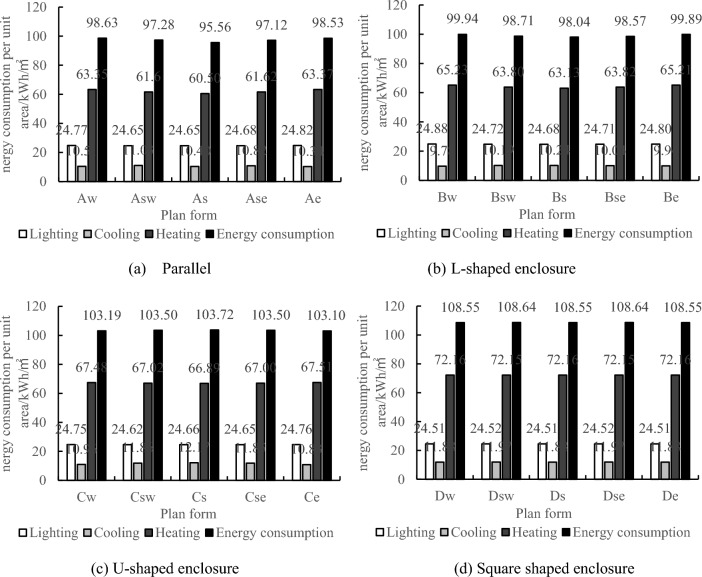
Energy consumption situations with a first-floor area of 2000 square meters.

From the annual total energy consumption, it can be observed that the orientation of the courtyard is an important factor affecting the energy consumption of exhibition buildings. According to Table 6, the parallel enclosure, designated as Type As, has the lowest energy consumption, which increases when the orientation deviates towards the east or west. The L-shaped enclosure, designated as Type Bs, has the lowest energy consumption when oriented towards the east or west, and increases when the orientation deviates from these directions. The U-shaped enclosure, designated as Type Cs, has the highest energy consumption, which decreases when the orientation deviates towards the east or west, with Type Ce having the lowest energy consumption among them. In the square enclosure, the west-facing window projection areas in Types Dsw and Dse are larger, resulting in higher energy consumption compared to Types Dw, Ds, and De. To further analyze the reasons for the variation in energy consumption with orientation, Fig. 7 is provided. The main reason for the decrease in energy consumption in Types As and Bs is the reduction in heating energy consumption during winter, with a decrease of approximately 5% and 3%, respectively. This indicates that for parallel and L-shaped enclosures, a south-oriented courtyard can better maintain indoor temperatures during winter and reduce heating energy consumption. For Type Ce, the main reason for the decrease in energy consumption is the reduction in cooling energy consumption during summer, with a decrease of approximately 11%, which is much greater than the 1% increase in heating energy consumption during winter. Therefore, an east-oriented courtyard is more suitable for U-shaped enclosures. The variation in cooling energy consumption during summer is greater for square enclosures, as they are more influenced by the western sun exposure. Types De, Ds, and Dw have lower energy consumption compared to Types Dse and Dsw, which have larger west-facing projection areas.

In conclusion, the parallel, L-shaped, and U-shaped enclosures are not suitable for placing the courtyard on the west side, while the square enclosure should minimize the window projection areas on the east and west orientations. The south-oriented courtyard in the parallel and L-shaped enclosures effectively reduces heating energy consumption during winter, while the east-oriented courtyard in the U-shaped enclosure significantly reduces cooling energy consumption during summer. Therefore, a south-oriented courtyard is more suitable for parallel and L-shaped layouts, while an east-oriented courtyard is more suitable for the U-shaped layout. Square enclosures with east, west, and south-oriented courtyards are more energy-efficient. These conclusions are applicable to exhibition buildings with a first-floor area ranging from 2000 to 13,000 m^2^, although the influence of orientation on energy consumption weakens as the first-floor area increases.

### Example verification: Liangzhu Culture Museum

**Figure 8:**
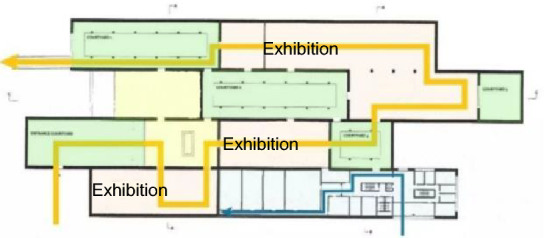
Functional blocks and flow chart of Liangzhu Culture Museum.

The Liangzhu Cultural Museum, completed in 2018, is located on the banks of the canal in the Liangzhu Cultural Zone in Hangzhou. The project occupies an area of approximately 46,595.9 m^2^, with an exhibition area of around 4000 m^2^ and a building height of 14.42 m. The specific functional areas are shown in Fig. 8, with the outdoor space highlighted in green. The first-floor area of the Liangzhu Cultural Museum is approximately 6700 m^2^, and the courtyard space occupies an area of about 2000 m^2^, which aligns with the previously mentioned nonlinear function relationship.

**Figure 9:**
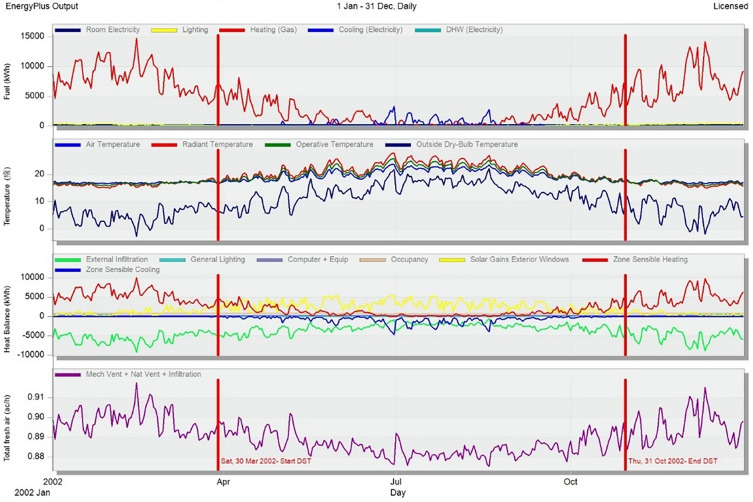
The energy consumption simulation results of the Liangzhu Cultural Museum.

However, in the actual operation of the museum, due to the excessive height of the Liangzhu Cultural Museum, the air conditioning system was installed at the top of the exhibition halls by the architects in order to achieve a clean indoor space. This not only resulted in inconvenience during the initial equipment installation phase but also led to a decrease in the cooling and heating effectiveness of the air conditioning system during the later operation, causing significant energy waste. After conducting performance simulation analysis of the building energy consumption of the Liangzhu Cultural Museum using DesignBuilder simulation software (Fig. 9), it was found that the heating and cooling energy consumption in the exhibition building during winter was high in the specific region. The specific values are shown in Table 9.

**Table 9:** Specific values of energy consumption of Liangzhu Culture Museum.

Energy consumption type	Total annual energy consumption/kWh	Total energy consumption per unit area/kWh/m^2^
Heating energy consumption	1,328,564.46	206.69
Cooling energy consumption	116,962.09	18.19
Lighting energy consumption	54,484.55	8.47
Internal electrical equipment	87,364.49	13.59
Total	1,587,375.59	246.94

**Figure 10:**
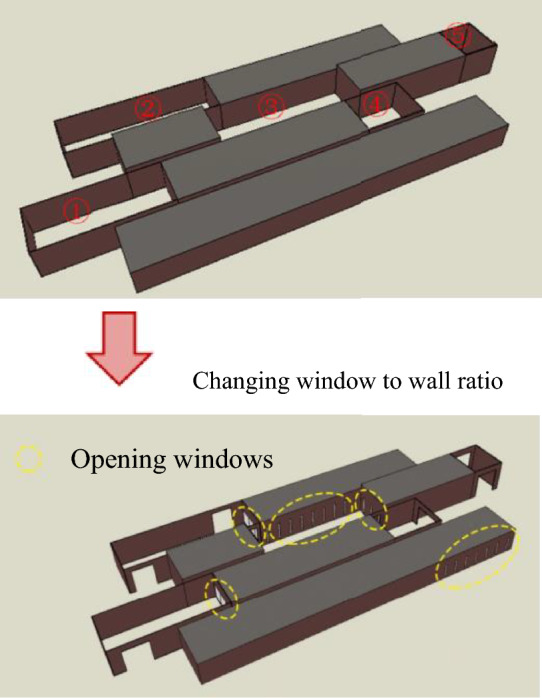
The before and after comparison of the form with optimized window-to-wall ratio.

To achieve the goal of a good indoor–outdoor interaction and low energy consumption in the Liangzhu Cultural Museum, the following optimization strategies are proposed from the perspectives of orientation and window-to-wall ratio, as shown in Fig. 10. Firstly, based on Fig. 4, the ground floor area of the Liangzhu Cultural Museum is 6700 m^2^. For Courtyards ①, ②, and ⑤, which are in a parallel layout, the optimal window-to-wall ratio for minimum energy consumption is between 38.7 and 39.2%. For the Square Enclosed Courtyard (③), the optimal window-to-wall ratio is between 29.1% and 29.8%. For the U-shaped Enclosed Courtyard (④), the optimal window-to-wall ratio is between 31.1 and 31.8%. In addition, considering the characteristic of exhibition halls not having opening windows and avoiding extensive west-facing windows, the window-to-wall ratio for the exterior walls of the exhibition halls and west-facing walls is set to 10%. Secondly, in terms of orientation, the long side of the building should preferably face south to minimize the impact of west-facing sunlight. Since the project has already been completed and changing the orientation would also affect the optimization of outdoor wind environment, efforts should be made to mitigate the corresponding energy consumption loss by adjusting the window-to-wall ratio. In future bidding and design processes, priority can be given to the consideration of orientation.

As shown in Table 10, the DesignBuilder software provides more intuitive data to reflect the changes in energy consumption before and after the optimization of the design. After the optimization, the annual heating energy consumption can be reduced by 92,999.51 kWh, the annual cooling energy consumption can be reduced by 12,865.83 kWh, the annual lighting energy consumption can be reduced by 1634.54 kWh, and the annual internal power equipment consumption can be reduced by 1747.29 kWh. The total energy savings for the year amount to 109,247.17 kWh, resulting in an energy saving rate of approximately 7.0%. Through calculations, it is estimated that 109,247.17 kWh is equivalent to the energy produced by the combustion of 13,437.06 kg of standard coal. If converted to smokeless washed anthracite coal (with a conversion coefficient of 0.900 0 compared to standard coal), it would be 14,930.07 kg. The market price of smokeless washed anthracite coal in China is approximately 970 yuan/ton. Therefore, after the design optimization, the annual energy cost can be reduced by approximately 14,482 yuan.

**Table 10:** Comparison of energy consumption before and after optimization.

Energy consumption type		Total annual energy consumption/KWh	Total energy consumption per unit area/KWh/m^2^	Annual energy savings/KWh
Heating energy consumption	After optimization	1,235,564.95	192.22	92,999.51
	Original solution	1,328,564.46	206.69	
Cooling energy consumption	After optimization	104,096.26	16.19	12,865.83
	Original solution	116,962.09	18.19	
Lighting energy consumption	After optimization	52,850.01	8.22	1634.54
	Original solution	54,484.55	8.47	
Internal electrical equipment	After optimization	85,617.2	13.32	1747.29
	Original solution	87,364.49	13.59	
Total	After optimization	1,478,128.42	229.95	109,247.17
	Original solution	1,587,375.59	246.95	

Annual energy savings = Total annual energy consumption Original solution − Total annual energy consumption after optimization.

## Discussion

### Scope and methodology of the study

Firstly, the sample size of well-known exhibition architectural cases from both domestic and international sources is crucial for the scientific validity of this research^8^. Considering the feasibility of data collection and on-site investigations during the actual research process^17^, this study primarily covers 48 exhibition architecture works, which are mainly designed by Pritzker Prize laureates and renowned architectural design teams. This approach aims to conduct a more comprehensive, in-depth, and reliable research analysis of exhibition architecture worldwide^12,22^. Similar related studies typically refer to a minimum of 30 cases to ensure the scientific rigor of data analysis. Therefore, based on the examination of 48 well-known exhibition architecture cases from both domestic and international sources, this paper proposes a nonlinear quadratic function relationship between the ground floor area and courtyard space area. This approach, based on “data experience”, offers architects a reference for exhibition architecture design. However, to validate the feasibility of the function itself, further support from additional case studies is required. To address this issue, in subsequent research, the author will continue to provide more reliable data support for this study through methods such as site visits, multi-channel data collection, etc.

Secondly, there are certain limitations regarding the scale and form characteristics of the research objects^25^. In the literature analysis, it can be observed that due to technical limitations of computer software and the difficulty in quantifying complex forms, many studies have resorted to simplifying architectural forms and summarizing typical layouts for simulation-based research^44^. In this study, we focus on the relationship between the ground floor area and courtyard area, primarily examining regular architectural forms with land sizes ranging from 10,000 to 50,000 square meters. This is because outdoor wind environment and building energy consumption are more likely to be influenced by such architectural typologies. However, specific research and exploration on complex architectural forms, irregular courtyard spaces, the number of courtyards, and elevated building levels have not been conducted yet, which will be the direction of future research. In our subsequent preliminary studies, we are excited to find that parameterizing layout factor parameters quantitatively and conducting quantitative simulation analysis of complex forms from a specific entry point will offer a fresh perspective on architectural design issues. This will provide designers with more practical design references that align with actual project requirements.

### Performance simulation and methods

Currently, many scholars have conducted research and exploration on building energy consumption and its influencing factors using energy simulation software such as DesignBuilder and Grasshopper. Some studies have shown that computer performance simulation methods play a certain role in reducing building energy consumption^9^. Additionally, some scholars have used PHPP software to simulate the energy consumption and analyze the economic costs of passive houses in summer hot and winter cold regions. Through this, they obtained energy consumption indicators for economically applicable passive houses in the local area and the range of thermal performance parameters for building envelopes^10^. Currently, the numerical simulation in this study is relatively simplified. In the performance simulation of energy consumption, factors such as the temperature of the building envelope, heat sources within the building, and economic cost analysis are highly complex during the use of exhibition buildings. These factors will be further explored in the next phase of research. In summary, both domestically and internationally, the performance design of exhibition buildings is still in a relatively blank stage^8,14^. The research in this paper on the layout forms and performance of exhibition buildings is only a preliminary exploration. It is hoped that in future research, the mentioned limitations and shortcomings can be addressed, and more emphasis can be placed on addressing the design issues of exhibition buildings from a quantitative rather than qualitative perspective. This will ensure that the research findings are more aligned with the actual needs of exhibition building design.

### The impact of window-to-wall ratio and orientation on energy consumption

In this study, energy consumption simulations were conducted on exhibition buildings with different window-to-wall ratios and orientations using DesignBuilder software. Numerical curves were obtained to illustrate the relationship between building energy consumption and window-to-wall ratio as well as orientation. In 2023, Fan et al.^45^ also used DesignBuilder as a simulation tool to compare the energy consumption of office buildings with different functional layouts in extremely cold, cold, summer hot and winter cold, and summer hot and winter warm regions under varying window-to-wall ratios. They explored the impact of window-to-wall ratio on office buildings with different core locations. In 2023, Lin et al.^46^ analyzed the influence of window-to-wall area ratio on building energy consumption in typical office buildings through energy consumption simulations. They found that, considering indoor lighting, a window-to-wall ratio of around 0.4 in the north–south direction provided the most optimized lighting effect and quality in terms of cost-effectiveness. Additionally, some scholars studied the impact of window-to-wall ratio on building energy consumption in Dalian using Grasshopper software^47^. The research indicated that an increase in window-to-wall area ratio led to an increase in cooling energy consumption and average energy consumption, while the change in heating energy consumption was related to window orientation, varying with different orientations. Therefore, numerous studies have shown that reasonable design of window-to-wall ratio and orientation in the early stages of architectural design can effectively reduce building energy consumption.

Furthermore, the author found that most scholars believe that there is an optimal value for window-to-wall ratio and orientation^16,30,46^, which provides the best energy-saving effect for buildings, aligning with the findings of this study. This research identified the minimum energy consumption window-to-wall ratio for exhibition buildings. When the ground floor area ≥ 9000 square meters, the minimum energy consumption window-to-wall ratio showed a stable trend, with ratios of 39.7%, 38.6%, 32.6%, and 30.6% for parallel, L-shaped, U-shaped, and square enclosures, respectively. In terms of building orientation, parallel and L-shaped enclosures were more suitable for south-facing courtyards, while U-shaped enclosures with the courtyard placed on the east side were preferable. Square enclosures require further analysis of the window projection surface on the west side. However, unlike other studies, this research focused more on the scientific rigor of the conclusions, with a precision adjustment of 0.1% for window-to-wall ratio and 45° for orientation. This approach provides more valuable design references for architects.

## Conclusions

This study evaluates the impact of courtyard orientation and window-to-wall ratio on the annual per unit area energy consumption of exhibition buildings, with a dual objective of establishing a good indoor–outdoor interactive relationship and achieving low energy consumption. The findings provide design references for the creation of exhibition buildings, and the following conclusions are drawn:

1. Under the climate conditions of the Yangtze River Delta region, the annual per unit area energy consumption of exhibition buildings exhibits a decreasing trend followed by an increasing trend as the window-to-wall ratio increases, indicating the presence of a minimum energy consumption window-to-wall ratio. Among them, the parallel enclosure has the highest minimum energy consumption window-to-wall ratio, while the square enclosure has the lowest ratio, with the order of arrangement being parallel > L-shaped > U-shaped > square.
2. When the ground floor area is less than 9000 square meters, the increase in the minimum energy consumption window-to-wall ratio becomes faster with an increasing ground floor area. When the ground floor area is greater than or equal to 9000 square meters, the change in the minimum energy consumption window-to-wall ratio shows a stable trend. The minimum energy consumption window-to-wall ratios for parallel, L-shaped, U-shaped, and square enclosures are 39.7%, 38.6%, 32.6%, and 30.6% respectively. Under this window-to-wall ratio condition, the annual per unit area energy consumption remains relatively constant as the ground floor area increases.
3. As the ground floor area increases, the impact of orientation on energy consumption decreases. Parallel and L-shaped enclosures are more suitable for south-facing courtyards, while U-shaped enc with the courtyard placed on the east side are preferable. None of the mentioned layouts are for a courtyard placed on west side. Analysis of the window projection on the west side is needed for the square enclosure, aiming to minimize energy consumption loss by reducing the window projection surface on the west side. In conclusion, this study provides a reference for the design of low-energy exhibition buildings with a good indoor-outdoor interactive relationship. It should be noted that this research still has certain limitations. The case study land sizes mentioned range from 10,000 to 50,000 square meters, and the forms are regular, without specific research and exploration on complex forms and multiple building clusters (such as segmented enclosures). Additionally, the square enclosure shows similar energy consumption results for east, west, and south orientations in the simulation results, but in actual projects, the situation becomes more complex due to the need for block interruptions and the establishment of elevated levels to ensure building use. This will be the direction of future research.

## Data Availability

The raw data supporting the conclusions of this article will be made available by the authors, without undue reservation. Contact the author Han Xinyu to get the data in this study.
